# Rupture of Uterus

**Published:** 1864-04

**Authors:** E. Holderness

**Affiliations:** Towanda, McLean Co., Ill.


ARTICLE XVI.

### RUPTURE OF UTERUS.

By E. HOLDERNESS, M.D., of Towanda, McLean Co., Ill.

Dr. N. S. Davis—*Dear Sir:*—Herein I send you the account of a case that may have some points of interest to your readers, if it is worthy of publication at all.

On the night of the 23d of February, I was called to see Mrs. D———. On my arrival, I found that she had had labor pains some half-hour previous, but they had ceased. During the few pains she had the waters broke. After waiting for a short time, I made examination per vagina, but could detect no presentation—the parts appeared to be relaxed, and in a pliable condition. From the time of my arrival (which was about 12 A.M.) until between 5 and 6 o'clock in the morning, she had no more pains, and appeared to rest easy. About this time (5 o'clock) pains commenced again, occurring at regular intervals, and I made another examination, but still could detect no presenting part. Her pains continued increasing in frequency and power, and between 6 and 7 o'clock, I detected the presentation to be that of the head, and it soon descended into the superior strait. Pains continued vigorous, and the head advancing slowly, but not sufficient for the amount of labor spent. I was satisfied I could feel the hairy scalp; to all appearances the membranes were broke, and that I could feel a soft substance, which I took to be one side of the placentia. I could not find either of the fontinelles, or any other feature to assure me of the position of the presentation. However, I did not see that I could do otherwise than wait patiently for a time, as there were no urgent symptoms, and see if the natural evolutions of the fœtus would not reveal something. The pains during the time had increased to the maximum of normal labors, but they appeared to accomplish but little. I found that nearly the whole force of the uterine efforts were expended upon the arch of the pubis; still the head had advanced some, and soon would have distended the soft parts. About 8 o'clock the pains commenced lagging, and I gave her a fluid dr. of wine ergot; but the pains continued flagging, with but little progress of the fœtus. Soon after 8 o'clock I noticed that she was either fainting or sinking, but could conceive of no reason why. Actual hard labor had not been of long duration, and little if any hæmorrhage had occurred. I had no instruments with me to facilitate delivery, and did not see that I could turn and deliver in that way. Seeing that the woman was in danger, I requested help, and Dr. BALLARD, of Bloomington, was sent for. The pains still continued, but were very short, terminating very abruptly—she was evidently sinking very fast. I used brandy, ammonia, and camphor, in hopes to produce reaction, but all to no purpose; she continued sinking, and soon died. Dr. BALLARD did not arrive until near 10 o'clock, and during the time I had returned home and gone into the country about one mile and a-half, and on my return home, learned that Dr. B. was at Mr. D.'s, and requested my attendance immediately. On my return to Mr. D.'s, I found that Dr. B. had just taken the child away (dead, of course,) without any trouble, saying that the presentation was all right.

When she died I expressed myself that death had been produced by internal hæmorrhage, but when Dr. B. took the child away, there was but a small quantity of blood escaped. Something else was amiss, but where, and what was it? The womb had contracted to the usual size after child-birth. We tried to detect the presence of fluid in the abdomen in the usual mode, but failed to detect any. I found that by placing the hand on the abdomen, and making a waving, up and down motion, we could detect the existence of fluid within. Upon this revelation we had reason to suspect laceration of the womb, and such did prove to be the case, when cutting through into the cavity of the abdomen, which was full of blood. The rent appeared to extend from the cervix to the fundus. The rent was on the left side.

During the time of labor I detected nothing in the character of the pains different from normal labors, but I did notice that she could not lay on the left side. She lay with the most comfort on her back.

The child proved to be very much deformed in the formation of its head; the skull was imperfect, having no frontal, and only a portion of the parietal bones, the occipital being nearly entire. That accounts for my being deceived with regard to the placential presentation. There was hardly any nose, and a very small mouth; the chin and forehead approximated closely. Where the right eye should have been, there was a growth much resembling a protuberant umbillicus, of at least an inch in length. The eye was forced over to the right, near an inch from its proper place. In the middle of the forehead there was a like protuberance, but not as long. The head was not of abnormal size.

I have been sorry since that we did not examine the uterine structures.

*Query.*—Can you give me any light? I saw nothing in the case to forewarn me of the mischief. I think the rent was gradual.

